# Equine vitiligo-like depigmentation in grey horses is related to genes involved in immune response and tumor metastasis

**DOI:** 10.1186/s12917-021-03046-x

**Published:** 2021-10-25

**Authors:** Thomas Druml, Gottfried Brem, Brandon Velie, Gabriella Lindgren, Michaela Horna, Anne Ricard, Gertrud Grilz-Seger

**Affiliations:** 1 Institute of Animal Breeding and Genetics, University of Veterinary sciences Vienna, Veterinärplatz 1, A-1210 Vienna, Austria; 2 Equine Genetics & Genomics Group, School of Life & Environmental Sciences, University of Sydney, Sydney, Australia; 3 Department of Animal Breeding and Genetics, Swedish University of Agricultural Sciences Uppsala, Uppsala, Sweden; 4 Livestock Genetics, Department of Biosystems, KU Leuven, Leuven, Belgium; 5 Department of Animal Husbandry, Slovak University of Agriculture in Nitra, Nitra, Slovakia; 6 GABI, INRAE, AgroParisTech, Université Paris-Saclay, 78350 Jouy-en-Josas, France; 7 Pôle Développement Innovation Recherche, IFCE, 61310 Gouffern en Auge, France

**Keywords:** Vitiligo, Lipizzan horse, GWAS, Melanoma, Tumor metastasis

## Abstract

**Background:**

In horses, the autoimmune disease vitiligo is characterized by the loss of melanocytes and results in patchy depigmentation of the skin around the eyes, muzzle and the perianal region. Vitiligo-like depigmentation occurs predominantly in horses displaying the grey coat colour and is observed at a prevalence level of 26.0–67.0% in grey horses compared with only 0.8–3.5% in non-grey horses. While the polygenetic background of this complex disease is well documented in humans, the underlying candidate genes for this skin disorder in horses remain unknown. In this study we aim to perform a genome-wide association study (GWAS) for identifying putative candidate loci for vitiligo-like depigmentation in horses.

**Methods:**

In the current study, we performed a GWAS analysis using high-density 670 k single nucleotide polymorphism (SNP) data from 152 Lipizzan and 104 Noriker horses, which were phenotyped for vitiligo-like depigmentation by visual inspection. After quality control 376,219 SNPs remained for analyses, the genome-wide Bonferroni corrected significance level was *p* < 1.33e-7.

**Results:**

We identified seven candidate genes on four chromosomes (ECA1, ECA13, ECA17, ECA20) putatively involved in vitiligo pathogenesis in grey horses. The highlighted genes *PHF11*, *SETDB2*, *CARHSP1* and *LITAFD*, are associated with the innate immune system, while the genes *RCBTB1*, *LITAFD*, *NUBPL*, *PTP4A1*, play a role in tumor suppression and metastasis. The antagonistic pathogenesis of vitiligo in relation to cancer specific enhanced cell motility and/or metastasis on typical melanoma predilection sites underlines a plausible involvement of *RCBTB1*, *LITAFD*, *NUBPL*, and *PTP4A1*.

**Conclusions:**

The proposed candidate genes for equine vitiligo-like depigmentation, indicate an antagonistic relation between vitiligo and tumor metastasis in a horse population with higher incidence of melanoma. Further replication and expression studies should lead to a better understanding of this skin disorder in horses.

## Background

Vitiligo-like depigmentation in horses occurs predominantly in animals of grey coat colour and is characterized by progressive patchy depigmentation of the skin around the eyes, muzzle and the perianal region. This skin disorder is caused by the loss of melanocytes that produce epidermal pigment. In humans, vitiligo affects skin areas mainly in the face, hands, feet, and genitals and is observed in 0.5–2.0% of the population [1]. The pathogenesis of human vitiligo is described as an interaction between intrinsic melanocyte defects, autoimmune mechanisms, and environmental and genetic factors [2]. Melanocytes from normal pigmented skin areas of vitiligo patients differ from those of healthy patients as they grow less efficient and exhibit structural defects [1, 3]. In addition to intrinsic abnormalities, it has also been shown that exogeneous oxidative stress factors can initiate a higher level of melanocyte destruction in vitiligo affected individuals [4]. The autoimmune hypothesis, which represents the leading model to explain the etiopathogenesis of vitiligo, rests upon studies that documented higher levels of melanocyte specific antibodies capable of destroying cultured melanocytes in vitro [5, 6]. Moreover, vitiligo affected skin is characterized by elevated infiltration of killer cells and inflammatory dendritic cells and the higher level of cytotoxic CD8^+^ T lymphocytes in blood and skin of vitiligo patients [7–9]. Throughout the last few decades, genetic factors associated with vitiligo and vitiligo-like skin disorders have been studied by the use of high throughput technologies like high-density SNP (single nucleotide polymorphism) genotyping and NGS (next generation sequencing) related methods [2, 10, 11]. In humans, more than 40 susceptible loci have been verified by means of genome-wide association studies (GWAS), further supporting established pathways involved in the pathogenesis of this skin disease. To date, the identified risk loci can be assigned to five groups: (a) human leukocyte antigen genes (HLA/MHC region); (b) immunoregulatory genes; (c) melanocyte related genes; (d) apoptotic and cytotoxic genes and (e) loci with unknown functions for vitiligo [2]. However, a ‘convergence theory ‘, which summarizes the combination of all known aetiologic factors that impact melanocyte viability in epidermal tissue has recently been proposed [12].

Although vitiligo has been the object of intensive genomic scientific research in humans [2, 10, 11, 13] the genetic background of this autoimmune disease in horses remains unknown. Currently, phenotypic animal models of vitiligo-like depigmentation have been described in horses [14], dogs [14], cats [14], pigs [1], chickens [1], and mice [1]. In horses, clinical studies were presented for twelve Gelderlands, nine Thoroughbreds, four Arabians, four Belgians, one Oldenburg, one Mecklenburg, and one Quarter Horse [15–20]. Further reports of vitiligo-like phenotypes were published for Arabian horses (Arabian fading syndrome) [21, 22] Pura Raza Espanola – P.R.E., Kladrubian, and Lipizzan horses. These horse breeds are characterized by medium to high allele frequencies of the grey coat colour associated *STX17* mutation, which is causative for progressive greying and involved in vesicle transport [23–26].

Population genetic studies providing heritability and genetic correlation estimates for vitiligo-like depigmentation and melanoma in grey horses have also been performed by Curik et al. [24], Hofmanova et al. [25], and Sanchez-Guerrero et al. [26]. In a total population of 11,436 P.R.E. horses (prevalence of vitiligo-like depigmentation ranged from 2.8–20.5%, whereas in 5.044 grey P.R.E. horses higher values from 3.6–49.8% were reported [26]. For 376 grey Kladrubian horses Hofmanova et al. [25] documented a prevalence of 26.0 to 67.0%, and in Lipizzans vitiligo-like phenotypes have been reported to occur in 39.9–50.0% of horses [24, 27]. In all studies an increase of vitiligo-like depigmentation with age was shown, with the largest increase observed between 5 and 7 years. Heritability estimates for this depigmentation phenotype ranged from 0.09 to 0.64. The higher estimates were calculated for samples with higher frequencies of the greying associated G-allele on the *STX17* locus (h^2^ of 0.64 in Lipizzans [24], h^2^ of 0.62 in grey P.R.E [26]., h^2^ of 0.35 in grey Kladrubian horses [25]). Genetic correlations between vitiligo-like depigmentation and melanoma were reported as between − 0.19 and 0.28, with the level of progressive greying yielding higher genetic correlations (0.48 to 0.67) to vitiligo-like depigmentation. Curik et al. [24] assigned a partial heritability to the *STX17* locus of 0.23, which reduced the polygenetic heritability from 0.64 to 0.41. These results clearly demonstrate that vitiligo-like depigmentation in horses occurs at higher levels in horse breeds selected for grey coat colour and that it is characterized by moderate to medium heritability thereby indicating the presence of environmental effects and/or polygenetic background.

Despite multiple investigations into vitiligo in horses, genomic studies on vitiligo-like depigmentation have yet to be conducted. As such, in this study we performed a genome-wide association study to identify susceptibility genes or loci associated with vitiligo-like depigmentation in a cohort of Lipizzan horses selected for grey coat colour.

## Results

### Prevalence and effects of coat colour loci on vitiligo-like depigmentation grade

**Fig. 1:**
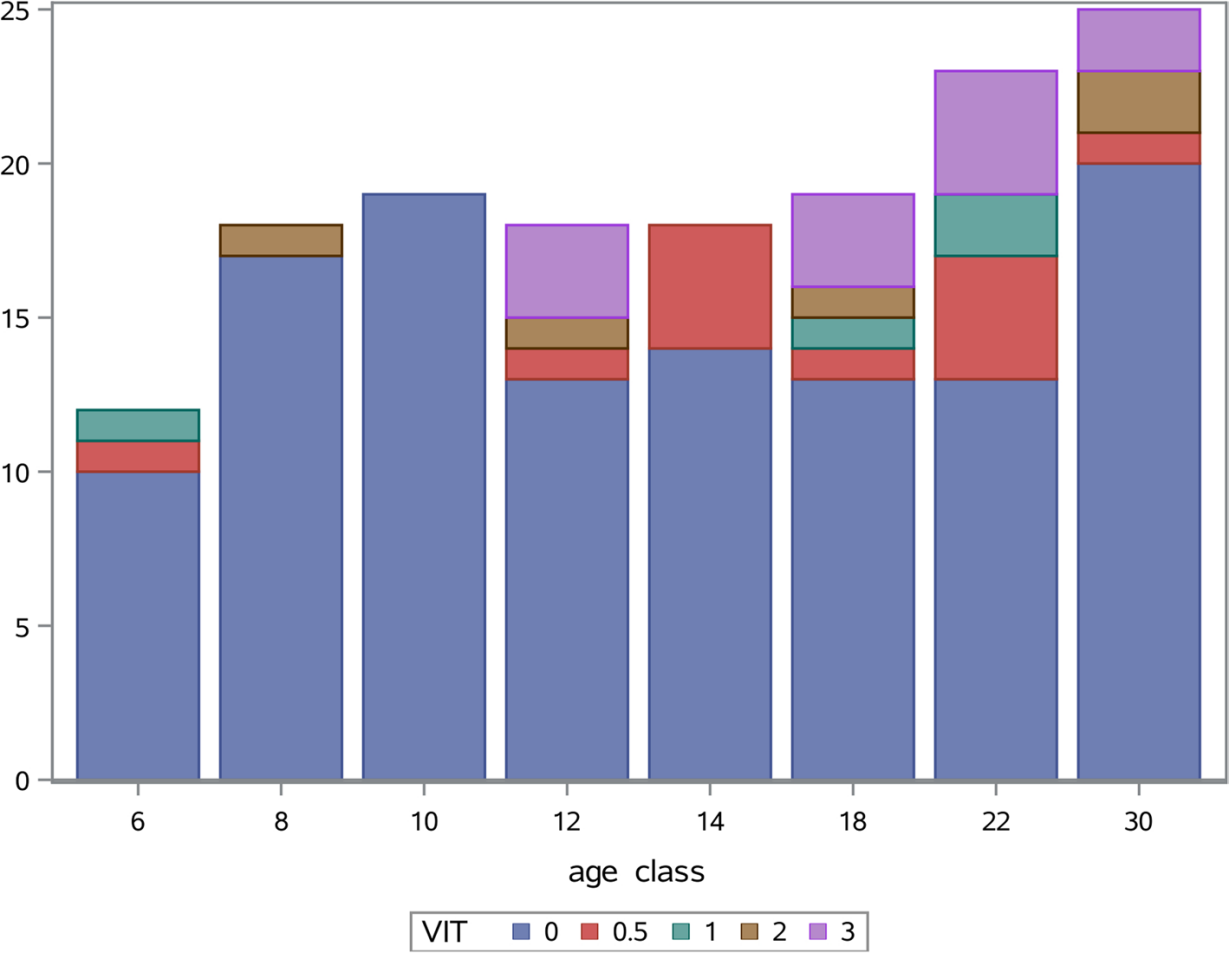
Distribution of 152 Lipizzan horses according to vitiligo-like depigmentation grade VIT (blue = grade 0; red = grade 0.5; green = grade 1; brown = grade 2; purple = grade 3) by age classes (class 6 = 4 to 6 years; class 8 = 7 to 8 years; class 10 = 9 to 10 years; class 12 = 11 to 12 years; class 14 = 13 to 14 years; class 18 = 15 to 18 years; class 22 = 19 to 22 years; class 30 = 23 to 34 years)

In the sample cohort of 152 phenotyped Lipizzan horses the vitiligo prevalence was 21.7% (33 horses from 152; Table 1). Phenotype distribution by age classes are presented in Fig. 1. From Table 1 and Fig. 1 a slight shift of higher vitiligo-like depigmentation towards higher individual age of horses can be observed, where 12 horses with grade 3 were on average 17.5 years old and 119 horses with grade 0 had an average age of 14.6 years (4 horses younger than 7 years were solid coloured).

**Table 1:** Observations, percentage, mean age and standard deviation of mean age (s.d.) for vitiligo-like depigmentation grade in 152 Lipizzan horses

Vitiligo grade	Observations	Percentage	Mean age	s.d. of age
0	119	78.29	14.56	7.38
0.5	12	7.89	16.08	5.57
1	4	2.63	16.00	6.73
2	5	3.29	17.80	8.90
3	12	7.89	17.50	4.64
	152	100	15.06	7.05

In Table 2 the genotype frequencies for the coat colour loci *ASIP, MC1R* and *STX17* within the sample cohort are given. From the coat colour genotype distribution only one chestnut (*MC1R* genotype *e/e*) was detected. Within the other Lipizzans the *MC1R* locus was nearly fixed for the dominant *E-*allele, which resulted in an allele frequency of 0.97. The allele frequency for the grey associated *G*-allele on *STX17* was 0.83 and 0.59 for the *A-*allele on *ASIP* locus. The sample included 12 Lipizzan horses of non-grey coat colour, which did not exhibit vitiligo-like depigmentation. The generalized linear model (GLM) including the fixed effects *ASIP*, *MC1R* and *STX17* genotype and age class, was able to explain 13.0% of observed variation in the dataset, whereas only the effect age class revealed a significant *p*-value of 0.046. The coat colour genotypes of *MC1R, ASIP* and *STX17* did not show an effect on vitiligo-like depigmentation in this sample of Lipizzan horses.

**Table 2:** Genotype distribution of *ASIP*, *MC1R* and *STX17* in 152 Lipizzan horses by vitiligo-like depigmentation grade

Vitiligo	***ASIP***			***MC1R***			***STX17***			
grade	***A/A***	***A/a***	***a/a***	***E/E***	***E/e***	***e/e***	***g/g***	***G/g***	***G/G***	Sum
**0**	34	64	21	112	6	1	12	30	77	119
**0.5**	2	6	4	12	0	0	0	0	12	12
**1**	2	2	0	3	1	0	0	0	4	4
**2**	3	2	0	4	1	0	0	1	4	5
**3**	6	6	0	12	0	0	0	4	8	12
**Sum**	47	80	25	143	8	1	12	35	105	152

### Genome-wide association analysis (GWAS)

**Fig. 2:**
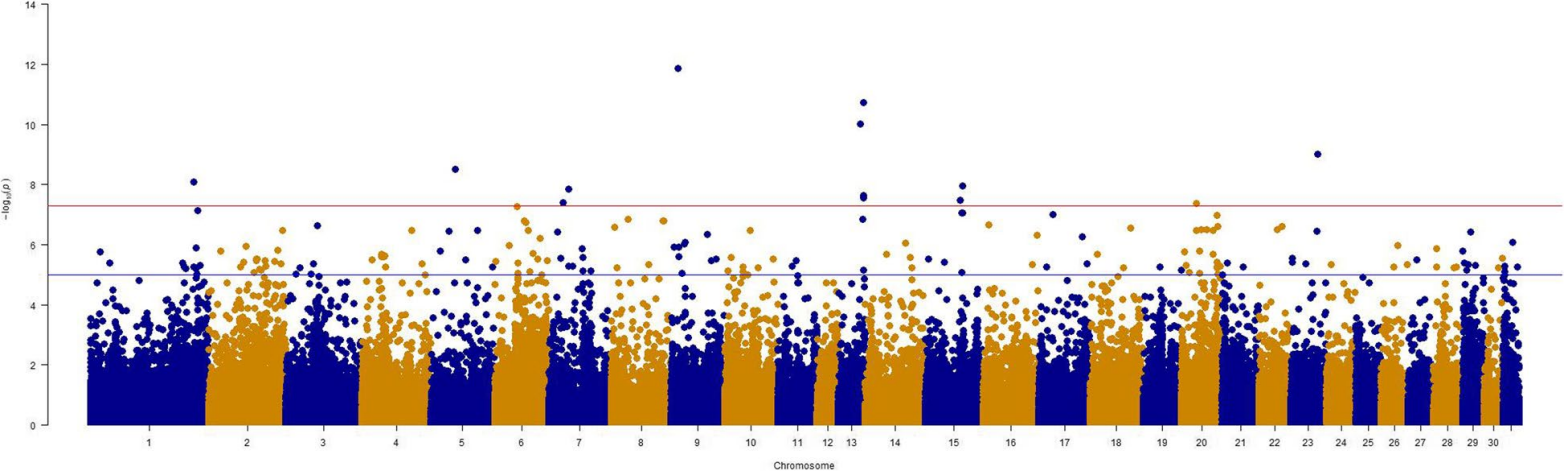
Manhattan plot of the GWAS for vitiligo-like depigmentation in 151 Lipizzan and 104 Noriker horses

The SNP-based heritability estimate for vitiligo-like depigmentation grade reached a level of 0.31 (s.d. 0.13). Genome-wide association analyses resulted in the detection of 17 genome-wide significant SNPs on 10 chromosomes (ECA1, 5, 6, 7, 9, 13, 15, 17, 20 and 23) (Fig. 2). Five SNPs were located within following genes: *NUBPL* (nucleotide binding protein like) on ECA1 (AX-104837465; *p* = 1.14e-07; intronic variant), *KCTD5* (potassium channel tetramerization domain containing 5) on ECA13 (AX-104387837; *p* = 1.88e-08; intronic variant), *PHF11* (PHD finger protein 11) on ECA17 (AX-104594172; *p* = 9.97e-08; intronic variant), *CARMIL1* (capping protein regulator and myosin 1 linker 1) (AX-103235989; *p* = 6.38e-08) and *PTP4A1* (protein tyrosine phosphatase 4A1) (AX-103697053; *p* = 1.06e-07; intronic variant) on ECA20.

From the significant SNPs, 8 SNPS were located in a window frame of +/− 25 kb containing one or two genes. These annotated genes were: *OR6N1* (olfactory receptor 6 N1), *OR6N2* (olfactory receptor 6 N2) on ECA5, *CLEC4D* (C-type lectin domain family 4 member D) on ECA6, *HNF4G* (hepatocyte nuclear factor 4 gamma) on ECA9, *LITAFD* (LITAF domain containing), *CARHSP1* (calcium regulated heat stable protein 1), *OR2C1* (olfactory receptor family 2 subfamily C member 1), *PDPK1* (3-phosphoinositide dependent protein kinase 1) and *AMDHD2* (amidohydrolase domain containing 2) on ECA13, and *RCBTB1* (RCC1 and BTB domain containing protein 1) and *SETDB2* (SET domain bifurcated histone lysine methyltransferase 2) on ECA17. Lastly, we identified 11 SNPs on eight chromosomes with annotated genes. Exact location, GWAS *p*-values, and gene annotation of the in total 17 significantly associated SNPs are given in Table 3.

**Table 3:** Significantly with vitiligo-like depigmentation associated SNPs including information on their position, *p*-values and annotated genes (genic = SNP located within gene; intergenic = SNP located in a +/− 25 kb window next to the gene)

Chr	SNP	rs SNP number	***p***-value	Position EquCab3	genic	gene region	genic SNP variant	intergenic -25 kb	intergenic + 25 kb
9	AX-104336590	rs68729604	1,18E-12	11,650,147					*HNF4G*
13	AX-104939678	rs1143810305	3,86E-11	40,644,649					*OR2C1*
13	AX-104150856	rs1145360759	1,88E-10	36,289,780				*LITAFD*	*CARHSP1*
23	AX-104329626	rs1148154226	1,14E-09	42,288,906					
5	AX-104234663	rs1138936192	3,13E-09	35,033,603				*OR6N2*	*OR6N1*
7	AX-104687221	rs1150319641	1,03E-08	32,228,883					
1	AX-103991294	rs1138560853	1,03E-08	164,725,011					
15	AX-103257076	rs1151004238	1,27E-08	59,455,614					
13	AX-104370576	rs1141793424	1,80E-08	41,503,474				*PDPK1*	*AMDHD2*
13	AX-104387837	rs68927375	1,88E-08	41,408,155	*KCTD5*	13:41387118–41,414,697	intronic (protein coding)		*PDPK1*
7	AX-103298246	rs1149149950	4,21E-08	23,815,408					
15	AX-104882695	rs69007837	6,30E-08	56,537,568					
20	AX-103235989	rs1147491448	6,38E-08	24,186,084	*CARMIL1*	20:23987962–24,299,401	intronic (protein coding)		
17	AX-104594172	rs1145363295	9,97E-08	21,505,125	*PHF11*	17:21489896–21,518,337	intronic (exon 5)	*RCBTB1*	*SETDB2*
20	AX-103697053	rs1148983319	1,06E-07	57,714,448	*PTP4A1*	20:57711442–57,718,044	intron 3		
6	AX-104673609	rs1143140285	1,09E-07	36,234,703				*CLEC4D*	
1	AX-104837465	rs1137469145	1,14E-07	170,926,834	*NUBPL*	1:170866249–171,111,362	intronic (protein coding)		

We further replicated the genotypes of the retained eleven SNPs with gene annotation within 1490 samples of the breeds Anglo-Arabian, Shagya-Arabian, Purebred Arabian, Partbred Arabian, Exmoor Pony, Selle Francais, Lipizzan, French Trotter, and Noriker. The sample of Lipizzan horses comprised 377 animals including Lipizzans from Slovak, Croatian and Hungarian stud farms and the samples of 174 Noriker horses contained animals of leopard spotting coat colour besides animals of the three basic colours bay, black and chestnut.

For the Anglo-Arabian, Purebred Arabian and Partbred Arabian samples we assumed according to breeding program definition a potential prevalence of 30–40% animals of grey coat colour. In Shagy Arabians 53% of animals were grey and within Noriker and Exmoor Pony grey coat colour does not segregate. In the breeds Selle Francais and French Trotter grey coat colour may occur at a low to moderate level (5–20%).

Out of the eleven SNPs, six loci (AX-104234663 (ECA5:35,033,603), AX-104673609 (ECA6:36,234,703), AX-104336590 (ECA9:11,650,147), AX-104387837 (ECA13:41,408,155), AX-104370576 (ECA13:41,503,474), AX-103235989 (ECA20:24,186,084)) did not show a meaningful genotype distribution, as higher proportion of genotypes associated with vitiligo-like depigmentation occurred at moderate level also in non-grey samples or samples where vitiligo-like phenotypes were not observed (Table 4).

**Table 4:** Distribution of SNP genotypes from eleven significantly associated SNPs with gene annotation within the breeds Anglo-Arabian, Shagya-Arabian, Purebred Arabian, Partbred Arabian, Exmoor Pony, Selle Francais, Lipizzan, French Trotter, and Noriker (NA = missing genotype)

		Anglo Arabian	Shagya Arabian	Purebred Arabian	Partbred Arabian	Exmoor Pony	Selle Francais	Lipizzan	French trotter	Noriker	Sum
**ECA1**	**AX-104837465**										
	**0**	7	32	155	21	273	295	315	156	171	1425
	**1**	0	0	0	0	0	0	46	0	2	48
	**2**	0	0	0	0	0	0	4	0	0	4
	**NA**	0	0	0	0	0	0	12	0	1	13
**ECA5**	**AX-104234663**										
	**0**	7	32	155	21	262	221	344	155	170	1367
	**1**	0	0	0	0	9	72	24	1	3	109
	**2**	0	0	0	0	1	2	0	0	0	3
	**NA**	0	0	0	0	1	0	9	0	1	11
**ECA6**	**AX-104673609**										
	**0**	6	32	152	20	61	241	243	126	173	1054
	**1**	1	0	3	1	141	51	102	28	1	328
	**2**	0	0	0	0	71	3	23	1	0	98
	**NA**	0	0	0	0	0	0	9	1	0	10
**ECA9**	**AX-104336590**										
	**0**	6	32	155	21	214	36	338	48	169	1019
	**1**	0	0	0	0	30	109	30	61	4	234
	**2**	0	0	0	0	0	77	0	15	1	93
	**NA**	1	0	0	0	29	73	9	32	0	144
**ECA13**	**AX-104150856**										
	**0**	7	16	126	18	265	287	361	155	174	1409
	**1**	0	14	28	3	1	7	15	0	0	68
	**2**	0	1	1	0	0	0	0	0	0	2
	**NA**	0	1	0	0	7	1	1	1	0	11
	**AX-104939678**										
	**0**	7	27	130	19	273	295	356	156	174	1437
	**1**	0	5	25	2	0	0	21	0	0	53
	**2**	0	0	0	0	0	0	0	0	0	0
	**AX-104387837**										
	**0**	7	25	104	17	262	254	353	113	171	1306
	**1**	0	7	47	3	11	41	23	37	3	172
	**2**	0	0	4	1	0	0	0	6	0	11
	**NA**	0	0	0	0	0	0	1	0	0	1
	**AX-104370576**										
	**0**	7	25	104	15	261	260	337	79	165	1253
	**1**	0	7	47	4	11	31	33	57	5	195
	**2**	0	0	4	1	0	1	0	20	0	26
	**NA**	0	0	0	1	1	3	7	0	4	16
**ECA17**	**AX-104594172**										
	**0**	7	28	155	21	273	286	333	155	168	1426
	**1**	0	4	0	0	0	9	33	1	6	53
	**2**	0	0	0	0	0	0	0	0	0	0
	**NA**	0	0	0	0	0	0	11	0	0	11
**ECA20**	**AX-103697053**										
	**0**	7	27	119	19	272	280	357	156	165	1402
	**1**	0	5	35	2	1	14	12	0	2	71
	**2**	0	0	1	0	0	1	4	0	0	6
	**NA**	0	0	0	0	0	0	4	0	7	11
	**AX-103235989**										
	**0**	5	32	61	12	226	234	233	77	166	1046
	**1**	0	0	66	4	47	39	103	62	4	325
	**2**	1	0	11	3	0	2	32	13	1	63
	**NA**	1	0	17	2	0	20	9	4	3	56
	**Sum**	**7**	**32**	**155**	**21**	**273**	**295**	**377**	**156**	**174**	**1490**

From the remaining five SNPs, the locus AX-104837465 (ECA1: 170,926,834), located within *NUBPL*, exhibited a nearly perfect association across breeds (46 heterozygous and 4 homozygous Lipizzans for the associated allele; 2 Noriker heterozygous for the associated allele), and SNP AX-104594172 (ECA17: 21,505,125), located within *PHF11*, showed a good, plausible, genotype distribution which included 33 Lipizzans, 6 Noriker, 9 Selle Francais, 4 Shagya-Arabians and 1 French Trotter, heterozygous for the vitiligo-like phenotype associated allele. The remaining three SNPs can be classified fair to good, as they indicate plausible associations, however exhibiting higher frequencies of the associated alleles among non-grey breeds.

**Fig. 3:**
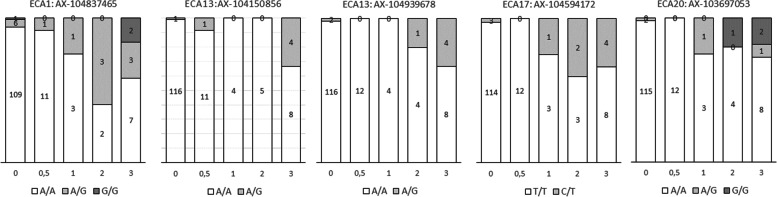
Distribution of SNP genotypes within phenotype classes of vitiligo-like depigmentation for the candidate SNPs ECA1: AX-104837465, ECA13: AX-104150856, ECA13: AX-104939678, ECA17: AX-104594172, and ECA20: AX-103697053

Figure 3 illustrates the genotype distributions of the resulting five plausible associated SNPs. Together they explain 38.5% of phenotypic variance of vitiligo-like depigmentation grade. Although no perfect association can be seen in the histogram plots, plausible distributions are documented for AX-104837465 on ECA1 (located within *NUBPL)*, AX-104594172 on ECA17 (located within *PHF11;* intergenic in a +/− 25 kb window of *RCBTB1* and *SETDB2)*, and for AX-103697053 on ECA20 (located within *PTP4A1*). In total, we propose the genes *NUBPL, PHF11, SETDB2, RCBTB1, PTP4A1*, and at a weaker replication level the genes *LITAFD, CARHSP1* and *OR2C1* as possible candidates for vitiligo-like depigmentation in grey horses.

## Discussion

The prevalence of vitiligo-like depigmentation in this study was 21.7%, and thus is in agreement with values cited in previous literature. 3.6 to 49.8% of grey P.R.E. horses [26], 26.0 to 67.0% of grey Kladrubian horses [25] and 39.9 to 50.0% of grey Lipizzan horses [24, 27] exhibit this depigmentation disorder. The SNP based heritability for vitiligo-like depigmentation was estimated as 0.31, a value which also corresponds with results published in pedigree-data based studies (h^2^ ranging from 0.35 to 0.64). While in the works of Curik et al. [24], Seltenhammer et al. [27] and Hofmanova et al. [25], all studied horses were of grey coat colour, the comprehensive data set of Sanchez-Guerrero et al. [26] allowed for the detection of vitiligo-like depigmentation in 0.8 to 3.5% 6392 non-grey horses. As a result, the prevalence of this depigmentation disorder in non-grey horses can be compared to human populations where the autoimmune disease vitiligo was observed in 0.5 to 2.0% of the population [1]. In addition to the higher incidence of vitiligo-like depigmentation, grey horses carrying the *STX17* mutation also exhibit a higher incidence of melanoma, which ranges from 46.1 to 50.0% in grey Lipizzans [24, 27], from 12.5 to 16.0% in grey Kladrub horses [25], and from 1.5 to 4.7% in grey P.R.E. horses [26]. In non-grey horses, Sanchez-Guerrera et al. [26] found a markedly lower melanoma incidence ranging from 0.7 to 0.9%. Clinical studies in humans have shown an up to threefold reduced probability of vitiligo patients developing melanoma [28, 29]. Furthermore, several vitiligo susceptibility genes in humans, particularly those expressed in melanocytes, have been detected to be involved in susceptibility to malignant melanoma, but with opposite effects [30]. This inverse relationship led to the suggestion that vitiligo may represent a process of immune surveillance against malignant melanoma [1, 31]. A similar relationship has been reported in Sinclaire miniature swine, where certain breeding lines were selected for a high melanoma incidence [32]. In this animal model up to 85% of individuals develop melanoma, from which 90% resolve spontaneously followed by a vitiligo-like depigmentation of skin and hair (destruction of melanocytes) in the tumor location [33]. Collectively, these findings in vitiligo research correspond with reported prevalence and negative genetic correlations (r from −0.34 to −0.09 [24, 26];) of vitiligo-like depigmentation and melanoma in horses.

Although early genetic segregation studies aimed to document the Mendelian modes of inheritance of human vitiligo, it rapidly became evident that this skin disorder represents a complex, multifactorial disease [31]. Currently, up to 322 genes related to vitiligo in humans have been addressed by 202 scientific articles, all of them incorporated in the VitiVar database [11] (www. http://vitivar.igib.res.in). A common criterion for complex disorders is a polygenetic background including multiple loci with minor effects - making it difficult to derive stable concordance across populations in replication or verification studies. In this GWAS study on vitiligo-like depigmentation in Lipizzan horses, we were able to identify a total of eleven SNPs within or next to 16 annotated genes. Considering the previously discussed differences in vitiligo prevalence between horse breeds of grey and breeds of non-grey coat colour, we examined which of the eleven annotated SNPs resulted in meaningful genotype distributions in breeds (a) selected for grey coat colour, (b) where grey coat colour occurs at low to medium level, and (c) where the grey associated *STX17* mutation is not segregating. This replication analysis resulted in five SNPs which led us to 9 different genes *NUBPL, PHF11, SETDB2, RCBTB1, PTP4A1*, *LITAFD, CARHSP1* and *OR2C1.*

Nucleotide binding protein-like (*NUBPL*), located on ECA1, is required for the assembly of human mitochondrial complex I, which contains 45 evolutionally conserved mitochondrial and nuclear encoded subunits. In addition to its central role for the mitochondrial respiratory chain, overexpression of *NUBPL* has been documented in melanoma and colorectal cancer tissue [34, 35]. Although the effect of this gene on melanoma is still unclear, Wang et al. [35] characterized *NUBPL* as a metastasis-related gene as they were able to show that the overexpression of *NUBPL* in colorectal cancer tissue was positively correlated with lymph node metastasis and advanced staging of this form of cancer. The authors found that *NUBPL* induces epithelial–mesenchymal transition through the activation of ERK signaling pathway, a pathway which promotes tumor metastasis. Uncontrolled growth is a necessary step for the development of all cancers and activating mutations of ERK pathway were also shown to be essential for melanoma development and progression [36]. However, the antagonistic pathogenesis of vitiligo in relation to cancer specific enhanced cell motility and/or metastasis on typical melanoma predilection sites (eyes, muzzle, perianal region) may underline a plausible involvement of *NUBPL* into the genetics of vitiligo-like depigmentation in horses.

The significant SNP on ECA17 was assigned to three different genes: *PHF11, SETDB2, RCBTB1.* Plant homeodomain zinc finger protein 11 *(PHF11)* and its neighboring gene SET domain bifurcated histone lysine methyltransferase 2 (*SETDB2*), are both expressed in cells of the innate immune system and are involved in chromatin remodeling or transcriptional regulation. Several studies associated polymorphisms of these genes with increased serum IgE levels and asthma, eczema, and atopic dermatitis in humans [37, 38]. In more recent studies, *SETDB2* has been linked to epigenetic concepts [39]. The histone modifier *SETDB2* was found to modulate adaptative resistance mechanisms in tumor development and in macrophage plasticity in inflammatory processes by regulating genomic stability and/or H3K9me3-mediated silencing of gene transcription [39, 40]. Due to their involvement in immune reactions, macrophagy, metastasis promotion, and adaptive resistance in melanoma [39], *PHF11* and *SETDB2* fit in the classification categories of vitiligo related genes proposed by Shen et al. [2] and Kundu et al. [12]. In close proximity to *PHF11* was the gene *RCBTB1*, which is located within a 25 kb window to the associated SNP on ECA17. Chronic lymphocytic leukemia deletion gene 7 *(RCBTB1,* also called *Clld7*) has been described as a general candidate tumor suppressor [41]. Zhou and Münger [42] investigated biological functions of *RCBTB1* in osteosarcoma cell lines and found that this protein is responsible for inhibited cell growth and decreased cell viability. The same authors detected an indication for activation of the DNA damage/repair pathway, as reduction of Clld7 in epithelial cells resulted in resistance to apoptosis.

Another gene that was pinpointed by GWAS and replication analysis was *PTP4A1,* located at ECA20. Protein tyrosine phosphatase type IVA 1 belongs to a group of three prenylated PTPs (PTP4A1/2/3), which support growth and migration of tumor cells [43]. Sacchetti et al. [44] were able to show that *PTP4A1* was highly expressed in fibroblasts of patients with the autoimmune disease systemic sclerosis. The tyrosine phosphatase PTP4A1 promotes transforming growth factor β (TGFβ) signaling in human fibroblasts through enhancement of ERK activity. A knockdown of PTP4A1 results in reduced ERK activation and correlates with reduced activity of the protooncogene SRC [44].

The genes *LITAFD, CARHSP1* and *OR2C1,* all located on ECA13, exhibited less specific replication across breeds and cases in GWAS. SNP AX-104150856 is located in a +/− 25 kb window between the genes *LITAFD* and *CARHSP1.* Calcium-regulated heat-stable protein 1 (*CARHSP1)* is reported to act as a tumor necrosis factor alpha (TNF-α) stability enhancer. As TNF-α is required for the control of infection and the subsequent immune response, it plays a central role for the host response to infection and injury [45]. *LITAFD* (LITAF domain containing) belongs to the CDIP1/LITAF family. Similar to *CARHSP1,* the gene *LITAF* also encodes a transcription factor regulating the gene expression of the inflammatory mediator TNF-α [46]. Considered as tumor suppressor in several cancer types, *LITAF* was also shown to be involved in immune response and autophagy [47]. For *OR2C1* no specific biological roles has been reported yet.

Overall, GWAS analysis of vitiligo-like depigmentation in the current study pinpointed seven genes with known biological role in the following fields: (a) immune response – *PHF11*, *SETDB2*, *CARHSP1*, *LITAF*; (b) tumor suppression – *RCBTB1*, *LITAF*; and (c) enhanced proliferation in metastasis – *NUBPL*, *PTP4A1*. While cancer related genes (b) and (c) may reflect the antagonistic relation between vitiligo and melanoma proposed by Spritz [30], the highlighted genes related to immune response represent classical features of the immune regulatory theory of vitiligo pathogenesis proposed by Shen et al. [2].

## Conclusions

Given the complex nature of the skin disorder vitiligo, the seven highlighted genes by GWAS of vitiligo-like depigmentation in grey horses need further verification by expression studies and replication studies across breeds. The current study indicates a relationship between this depigmentation phenotype and melanoma in grey horses and represents a research question that needs further investigation.

## Material and methods

### Sample cohort

**Fig. 4:**
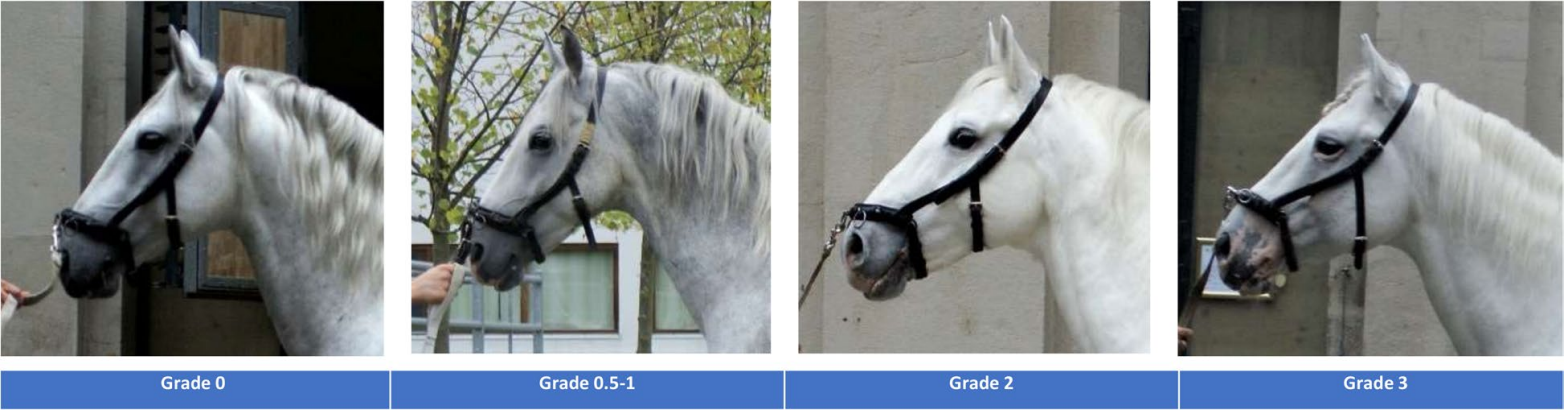
Phenotypic classification of vitiligo-like depigmentation

In the year 2020, 152 Lipizzan horses (60 mares and 92 stallions) from the Austrian federal stud farm Piber and the Spanish Riding School Vienna were phenotyped for vitiligo-like depigmentation by visual inspection according to the protocol of Curik et al. [24]. The following grades of vitiligo-like depigmentation were recorded: grade 0 = no depigmentation; grade 0.5 = beginning of non-segmental depigmentation via few small spots in the muzzle, perianal region; grade 1 = clearly visible non-segmental depigmentation in muzzle and perianal region; grade 2 = extended distribution of non-segmental depigmentation in face and eventually a few small segmented depigmentation areas, grade 3 = prevalence of extended sharp segmented depigmentation areas around muzzle, eyes and face (Fig. 4). The age of studied horses ranged from 4 to 34 years, with a mean age of 15.06 years (s.d. + 7.05), whereas 140 horses of the sample were older than 6 years, thus taking the etiology of this skin disorder into account [24, 25]. For all phenotyped horses, genotype data was available from previous studies [48–50] and this study was performed with the permission by the owner (Spanische Hofreitschule und Lipizzanergestüt Piber GöR). Blood/hair samples from these horses were collected under appropriate terms regarding ethical approvements (Commission for Ethics and Animal Welfare, University of Veterinary Medicine, Vienna, protocol number: ETK-06/05/2015, in accordance with GSP guidelines and national legislation) and were genotyped using the Axiom Equine Genotyping array (MNEc670k, Affymetrix, Inc., Santa Clara, CA, USA [51];).

### SNP quality control

As vitiligo-like depigmentation in grey horses develops with age [24, 25], we can assume that in a certain number of young horses, this depigmentation disorder may not yet be visible. In order to overcome this problem, we included 104 solid coloured horses from the Noriker breed without vitiligo-like characteristics as a control group. Quality control (QC) of the sample cohort consisting of 152 Lipizzan and 104 Noriker horses was performed using the software PLINK 1.07 [52]. SNPs located on sex chromosomes (X: 28,017 SNPs and Y: 1 SNP) and 30.864 SNPs without chromosomal assignment were excluded from further analysis. We further applied a filter for genotyping call rate for individuals of min. 95% and retained SNPs with a minor allele frequency larger than 1%, which resulted in 376,219 SNPs and 255 horses (104 Noriker, 151 Lipizzans) that passed QC.

### Determination of *MC1R*, *ASIP* and *STX17* genotypes and statistical analysis

In order to test the putative effects of the coat colour genes *ASIP*, *MC1R* and *STX17* on the vitiligo phenotype data, we used the 670 k SNP data to derive the genotypes of respective loci. For *MC1R* the causative point mutation is directly contained by the SNP AX-104805525 (ECA3:36,259,552) in the Affymetrix® Axiom Equine HD Array. From previous studies [49] the *ASIP* genotype was known for 118 horses. In a recent study, Corbin et al. [53] used the SNP AX-103951024 (ECA22: 24,877,990) to tag the *ASIP* deletion [54]. However, the respective SNP genotypes of the 152 analyzed Lipizzans were monomorphic, thus we correlated five SNPs (AX-103929593, ECA22:25,147,724; AX-103057353, ECA22:25,148,247; AX-102968633, ECA22:25,161,695; AX-104134604, ECA22:25,175,554; AX-103402623, ECA22:25,176,678; AX-103271875, ECA22:25,187,112) around the *ASIP* locus (ECA22:25,165.083-25,173,072) with the known *ASIP* genotypes of 118 Lipizzan horses derived from KASP (competitive allele specific PCR) analysis according to Rieder et al. [54]. The highest correlation (r = 0.97) was found for the SNP AX-102968633, whereas the correlation of the other four SNPs ranged between 0 and 0.21. Four observations from 118 did not match the KASP results, implying an error rate of 1.15 horses for the SNP derived genotype of 34 horses missing the *ASIP* genotype information. The *STX17* genotype was known for 130 Lipizzan horses from the study of Grilz-Seger et al. [49] by genotyping the causative 4.6 kb duplication in intron 6 of syntaxin 17 following the method of Kavar et al. [55]. In the work of Grilz-Seger et al. [38] the authors demonstrated that the *STX17* locus was surrounded by a selection signature, a 350 kb ROH (run of homozygosity) haplotype, that was shared by 70% homozygous grey horses. In order to derive the 22 missing *STX17* genotypes, we performed a ROH analysis for all 152 horses in our sample using the software PLINK 1.07 [52] and the following setting: minimal SNP density of one SNP per 50 kb, maximal distance between two homozygous segments of 100 kb, minimum length of homozygous segment of 80 kb and/or 20 consecutive SNPs per segment, one heterozygote and one missing SNP were allowed. The resulting ROHs were assigned to *STX17* genotypes as follows: (a) coloured Lipizzans – *STX17* genotype *g/g*; (b) grey Lipizzans with ROH haplotype around *STX17* – genotype *G/G*; (c) grey Lipizzans without ROH around *STX17* – genotype *G/g*. Resulting ROH genotypes of 130 horses were correlated with known *STX17* genotypes from fragment genotyping analysis, revealing a correlation of 0.89 (8 from 130 genotypes were wrongly assigned, implying that 1.3 horses out of 22 horses with unknown *STX17* genotypes can be wrongly estimated.

To test the putative influence of the factors *ASIP*, *MC1R* and *STX17* genotype, and age class on vitiligo-like depigmentation grade, we applied the following generalized linear model (GLM):

$${\mathrm{Y}}_{\mathrm{i}\mathrm{jklm}}=y+\mathrm{agre}\_{\mathrm{class}}_{\mathrm{i}}\kern0.5em {\mathrm{ASIP}}_{\mathrm{i}}+\kern0.5em \mathrm{MC}1{\mathrm{R}}_{\mathrm{k}}+\kern0.5em \mathrm{STX}{17}_1+\kern0.5em {\mathrm{e}}_{\mathrm{i}\mathrm{jklm}}$$

where:

Y_ijklm_ = observations.

*y* = mean.

age_class_i_ = age effect in age classes (6: 4 to 6 years, 8: 7 to 8 years; 10: 9 to 10 years; 12: 11 to 12 years; 14: 13 to 14 years; 18: 15 to 18 years; 22: 19 to 22 years; 30: 23 to 34 years).

ASIP_j_ = effect of ASIP genotype (*A/A, A/a, a/a*).

MC1R_k_ = effect of MC1R genotype (*E/E, E/e, e/e*).

STX17_l_ = effect of STX17 genotype (*G/G, G/g, g/g*).

e_ijklm_ = residual error.

Statistical analyses and graphical representations were performed using the software package SAS [56], SNP data management was done with PLINK 1.07 [52].

### GWAS analysis and replication study

GWAS analysis and heritability estimation for vitiligo-like depigmentation grade were performed using the software GCTA v. 1.91.6 [57]. The GWAS was carried out using the mixed linear model association (−mlma) method, thus taking genetic structure and individual relationship within the data into account:

$${Y}_{ij}={b}_j{SNP}_{ij}+{g}_i+\kern0.5em e\sim N\left(0,I{\sigma}_{e^2}\right)$$

where y_ij_ was the phenotype of the _i_th individual, b_j_ was the allele substitution effect of the _j_th SNP marker, SNP_*ij*_ was the genotype of the _i_th animal for the _j_th SNP, g_i_ was the random polygenic effect of the _i_th individual, and e_ij_ was the random residual effect for the _i_th individual and _j_th SNP. The polygenic effects (g) followed a normal distribution g ~ N(0, Gσ_g_^2^), where G was the genomic relationship matrix (calculated as described by Yang et al. [57], and the residuals followed a normal distribution e ~ N(0, Iσ_e_^2^). The *STX17* genotype was included in the GWAS as a covariate. Heritability for vitiligo-like depigmentation grade was estimated by the –reml command in the GCTA software [57].

Adjustment for genome-wide multiple testing of association statistics was carried out according to the Bonferroni method, which adjusts the *p* value threshold from *p* = 0.05 to p = 0.05/k, where k is the number of SNPs (376,219) in the GWAS, thus reaching a threshold of *p* < 1.33e-7. Manhattan plots and quantile-quantile plots were generated in R (www.r-project.com) using the package qqman.

For the identification of potential candidate genes for equine vitiligo-like depigmentation, we applied a two-step procedure, which is described as follows: step 1 – gene annotation: We screened the genome in a +/− 25 kb window frame surrounding each significantly associated SNP in the Ensembl genome database EquCab3.0 (www.ensembl.org). In the event that a SNP was located between two genes within the defined window frame, both genes were selected; step 2 – replication study of significant SNPs with gene annotation. We screened eight horse breeds for genotype distribution of the identified significant SNPs with gene annotation. With this replication study we aimed to determine which of the SNPs were segregating ubiquitously or specific in populations with: (a) higher *STX17* G-allele frequency (Lipizzan, Arabian populations); (b) where *STX17 G*-allele is not segregating (Exmoor Pony and Noriker); and (c) where *STX17 G*-allele can occur at low to moderate frequency (Selle Francais and French Trotter). The HD 670 k SNP genotype data set was comprised of 1490 samples from 377 Lipizzan horses, 174 Noriker horses, 32 Shagya Arabians, 155 Purebred Arabians, 21 Partbred Arabians, 7 Anglo-Arabians, 273 Exmoor Ponies, 295 Selle Francais, and 156 French Trotters. This data had already been published in Grilz-Seger et al. [50] and was used for this replication study by consent of the authors.

## Data Availability

The primary data of this study are owned by different research groups. Primary data of the breeds Lipizzan, Noriker, Shagya Arabian are available from project consortium FFG project number 843464, Veterinary University Vienna, Xenogenetik, five European state stud farms and the Austrian Horse breeders Association, but restrictions apply to the availability of these data, which were used under license for the current study, and so are not publicly available. Data are however available from the authors upon reasonable request and with permission of project consortium, FFG project number 843464, Veterinary University Vienna, Xenogenetik and partners. Genotype data for the Exmoor Pony breed can be provided by contacting authors Lindgren/Velie or for a larger data set via the following reference: Velie, B.D.; Shrestha, M.; Franҫois, L.; Schurink, A.; Tesfayonas, Y.G.; Stinckens, A.; Blott, S.; Ducro, B.J.; Mikko, S.; Thomas, R.; Swinburne, J.E.; Sundqvist, M.; Eriksson, S.; Buys, N.; Lindgren, G. Using an inbred horse breed in a high density genome-wide scan for genetic risk factors of insect bite hypersensitivity (IBH). PLoS One. 2016, 11, e0152966.
